# Neuropsychological Correlates of Pre-Frailty in Neurocognitive Disorders: A Possible Role for Metacognitive Dysfunction and Mood Changes

**DOI:** 10.3389/fmed.2017.00199

**Published:** 2017-11-15

**Authors:** Martina Amanzio, Sara Palermo, Milena Zucca, Rosalba Rosato, Elisa Rubino, Daniela Leotta, Massimo Bartoli, Innocenzo Rainero

**Affiliations:** ^1^Department of Psychology, University of Turin, Turin, Italy; ^2^European Innovation Partnership on Active and Healthy Ageing, Brussels, Belgium; ^3^Department of Neuroscience “Rita Levi Montalcini”, University of Turin, Turin, Italy; ^4^Unit of Cancer Epidemiology, “Città della Salute e della Scienza” Hospital and CPO Piemonte, Turin, Italy; ^5^Neurology Division, Martini Hospital, Turin, Italy

**Keywords:** pre-frailty, mild cognitive impairment, comprehensive geriatric assessment, Alzheimer’s disease, executive dysfunction

## Abstract

**Background:**

Recent studies have suggested that cognitive functions in patients with neurocognitive disorders have a significant role in the pathogenic mechanisms of frailty. Although pre-frailty is considered an intermediate, preclinical state, epidemiological research has begun to dislodge cognition and frailty into their specific subcomponents to understand the relationship among them. We aim to analyse the possible association between pre-frailty and neuropsychological variables to outline which factors can contribute to minor and major neurocognitive disorders.

**Methods:**

60 subjects complaining of different cognitive deficits underwent a deep-in-wide frailty and neuropsychological assessment. We conducted three multiple linear regression analyses adjusted for a combination of demographic measures and involving several neuropsychological–behavioural parameters selected by the literature on physical frailty.

**Results:**

We found a significant association between frailty—as measured by the multidimensional prognostic index (MPI)—and action monitoring and monetary gain (cognitive domain), depression and disinhibition (behavioural domain). Moreover, an association between MPI and impaired awareness for instrumental activities disabilities exists.

**Conclusion:**

We propose a novel framework for understanding frailty associated with metacognitive–executive dysfunction.

## Introduction

Frailty is a complex and heterogeneous clinical syndrome. It is described by augmented vulnerability resulting from age-related decline across several body organs and physiological systems (1, 2). Frailty leads to a decrease in the ability to remain independent and to maintain a good quality of life (3). Moreover, it increases the risk of disability and mortality (3). The frailty syndrome has principally been explored by focussing on the physical domain, mostly relying on signs and symptoms such as muscle weakness, sedentary behaviour, slow gait speed, and weight loss (1). Frailty has also been involved in general cognitive decline and impaired global cognition (4).

Canevelli and colleagues (5) have recently emphasised some criticalities of these studies: (a) the overwhelming majority of them have assessed frailty using the criteria proposed by Fried and colleagues (1), primarily aimed at describing the physical dimension of frailty; (b) most of these researches adopted only a measure of global cognitive functioning, mostly assessed by the Mini Mental State Examination [MMSE; (6)] to assess cognition, in absence of a comprehensive neuropsychological evaluation; (c) finally, the sample populations were mainly composed of community-dwelling older adults, preventing the applicability of results to other types of patients. The limitations of those studies have also been previously reported (7).

The overall concept of examining the specific cognitive correlates of frailty is interesting and may indeed shed light on a more comprehensive model of frailty that details a specific neuropsychological profile. Interestingly, the most novel evidence comes from a small number of studies aimed at examining the association between specific cognitive functions and physical frailty (5). In particular, Canevelli and colleagues (5) pointed out a significant impairment of attention and executive functions. Importantly, literature has shown that the executive function and attention domains are related to frailty, while gait speed or grip strength are the components of frailty most strongly associated with cognition (8–20). O’Halloran and colleagues (8) recently stated that pre-frail and frail individuals seem less able than robust subjects in the “Sustained Attention to Response Task” (SART). Even if these studies represent a first important attempt to describe the association between cognitive functions and physical frailty, there is still the need to assess frailty with a multidimensional approach (21–23).

Although executive function and attention domains may be related to physical frailty, no previous studies have investigated the role of different neuropsychological domains by carrying out a multidimensional assessment of frailty. In line with this, the multidimensional prognostic index (MPI) currently represents one of the suggested methods for a comprehensive assessment of frailty (24–26).

The aim of this study is to investigate whether pre-frailty, measured through the MPI, might be influenced by cognitive–behavioural measures in individuals with minor and major neurocognitive disorders [DSM-5; (27)], from mild cognitive impairment (MCI) likely due to Alzheimer’s disease (AD), to mild AD patients (28). For this reason, we conducted three multiple linear regression analyses to study: (1) the role of global cognitive functioning and specific cognitive variables (selective attention, episodic memory, language comprehension, and reasoning in the visual modality); (2) the role of metacognitive executive functions, such as response monitoring; and (3) the relationship with mood changes, quality of life, and awareness of independence for daily living instrumental activities.

## Materials and Methods

### Participants

All the outpatients were enrolled at the Neurology Division of the “Città della Scienza e della Salute” Hospital and the Martini Hospital, both in Turin (Italy).

Participants were included in the study if they had: (a) minor or major neurocognitive disorders (27), such as MCI-likely due to AD and mild AD. On the other hand, participants were excluded from the study if they had: (a) dysthymia or major depressive disorder, based on DSM-5 criteria (27); (b) A MMSE score <20, given that the neuropsychological measurement is not as reliable when problems of language comprehension occur; (c) were taking medications that could substantially impact cognitive functioning. CSF diagnosis that did not provide *in vivo* evidence of Alzheimer’s pathology was considered exclusion criterion.

The patients underwent a complete neurological examination and neuroradiological investigations (MRI and FDG-PET). All the patients underwent lumbar puncture with cerebrospinal fluid measurement of 1-42 beta-amyloid, phospho-Tau, and total-Tau (Innogenetics kits, Ghent, Belgium).

### Assessment of Frailty

Several operational definitions have been proposed to assess frailty in the elderly (29). Although this variability might be acceptable from a public health perspective, the identification of a gold standard measure might still be important to obtain. To date, frailty is still detected using different instruments and none of them has been able to prevail as superior to the others (29).

An index originally conceived and designed in the Italian general practice setting for the evaluation of chronic multipathological conditions was initially used to screen the participants’ levels of frailty (30). The scale for the Evaluation of the frailty elderly (Scheda di Valutazione dell’Anziano Fragile,^1^ SVAFRA) is a “multiaxial” score, interpreted according to the principles of the complexity theory in medicine and healthcare (30). This scale is a useful tool in case of multiaxial assessment in the setting of primary care (31), especially for its ability to combine technical skills, synthesis of information, and care-management suggestions (30, 31). SVAFRA was used for the first “Cognitive survey on the frailty of elderly people in Italy,” which was attended by 34 Primary Care Physicians and 521 elderly recruited in Veneto, Lazio, and Sicily (32).

According to the authors, frailty is determined by issues pertaining at least one of the following evaluative dimensions (30): physical and mental health; physical and cognitive–behavioural disabilities; patient management considering the care burden; family and socio-environmental background. For each axis, determinants can assume a value between 0 (no deficiencies) and 3 (very serious problems). The level of frailty is then classified based on a combination of these four dimensions. A synthetic value—regardless of the value previously assigned to the four determinants—can also be assigned: F0 (absence of frailty, typical of healthy patients); F1 (pre-frailty); F2 (medium frailty); F3 (severe frailty, typical of severely ill patients); T (extreme frailty, typical of *terminally ill* patients).

Since clinicians needed an outcome instrument with sound clinimetric properties (33), frailty was assessed using a comprehensive geriatric assessment (CGA), which was considered the first-choice tool for evaluating biological, functional, cognitive, social, and clinical aspects of elderly subjects, as expressed by the Italian Society of Gerontology and Geriatrics (34, 35). The MPI was developed from a standardised CGA (26, 35, 36). It was originally conceived as a prognostic index of mortality in the short- and long-term period based on information obtained through the CGA in hospitalised (37) and outpatient settings (38). With this index, the prognostic value of negative outcomes and related severity are even higher than with the indices calculated in the single domains of the rating. The MPI is also considered suitable for assessing frailty in the elderly (34–36). Moreover, as a cumulative multidimensional index, the MPI allows professionals to develop a treatment plan (22). In 2013, the European Innovation Partnership on Active and Healthy Ageing identified 98 “good practices” involved in research to reverse or prevent frailty (24). As demonstrated by the Marco Polo Initiative, the MPI is helpful in identifying frailty elders, providing personalised interventions, avoiding drug prescriptions and adverse hospital admissions (24, 39).

The MPI includes information on clinical, functional, nutritional, and neuropsychological aspects, as well as polypathology, pharmacological treatment, and the social support network (22, 25, 26, 35–40). Specifically, the MPI is calculated using a mathematical algorithm that includes scores of individual profiles obtained from the eight evaluation tools that make up the CGA: 1. *activity of daily living scale* (ADL); 2. *instrumental activity of daily living* scale (iADL); 3. *short portable mental status questionnaire* (to assess cognitive status); 4. *cumulative illness rating scale—comorbidity index* (CIRS-CI, to explore comorbidity); 5. *Mini Nutritional Assessment* scale (MNA, to assess nutritional status); 6. *Exton Smith Scale* (to evaluate the risk of developing pressure sores); 7. polypharmacy; 8. social condition (22, 25, 26, 35–40).

The numerical index obtained has a value between 0.0 (lowest risk) and 1.0 (highest risk of mortality) and reports three grades of risk of severe prognosis: low risk (MPI value ≤ 0.33), moderate risk (MPI value between 0.34 and 0.66), and severe risk (MPI ≥ 0.67) (22, 26, 36, 37, 40). Importantly, the effectiveness of the MPI has recently been verified in population-based cohorts. Higher MPI risk scores were associated with more days in hospital and with fewer years of survival, across a broad and stratified age range (25, 40).

### Neuropsychological Assessment

The neuropsychological evaluation involved a wide assessment of global cognitive deterioration using: the CDR (41), the Addenbrooke’s Cognitive Examination-Revised version [ACE-R; (42)] and, finally, the MMSE (6). Other cognitive domains were also assessed with the use of different scales: selective attention with Attentional Matrices [AM; (43)], divided attention and cognitive shifting with the Trial Making Test [TMT; (43, 44)], episodic memory with the Recall of a Short Story test [Babcock; (37)], reasoning in the visual modality with Coloured Progressive Matrices [CPM-36; (43)], comprehension of spoken language with the Token Test [TT; (43, 45)].

Executive functions were analysed with the metacognitive version of the Wisconsin Card Sorting Test [m-WCST; (46, 47)]. This test is aimed to assess “on-line” metacognitive monitoring and control during the execution of the test (46, 47). For each card of the test, two questions evaluated “on-line” metacognitive monitoring (“What is your degree of confidence in this answer?”) and control (“Do you want to take this response into account in your total score?”) (46–48). In the original version of Koren’s protocol, the participants were assigned a dollar value for each correct response (46–48). In this study, the patients received a monetary gain of 10 cents for each correct answer and they were deprived of 10 cents for every wrong answer (49). As described in our previous work that used the same procedure (49), a set of metacognitive indices were evaluated: “(1) accuracy score (AS), the number of correct voluntary answers/number of voluntary responses; (2) free choice improvement (FCI) (accuracy—number of correct responses from forced responses)/number of cards presented; (3) global monitoring (GM), the number of correct responses—the total number of sorts required in the final score; (4) monitoring resolution (MR), the gamma correlation calculated between the confidence and correctness of the sorts in the entire test; (5) control sensitivity (CS), to what extent the control process depended on the monitoring process, indexed by the gamma correlation calculated across all sorts between the level of confidence and the decision to gamble; (6) monetary gains (MG), given by the number of correct voluntary responses—incorrect number of voluntary responses” [(49), p. 138].

We verified the subjects’ level of autonomy in basic and instrumental activities of daily living (50, 51) and accordingly, their quality of life with the Quality of life scale in Alzheimer’s disease (QoL-AD)—patient’s subscale (52). Patients were also assessed using specific neuropsychiatric rating scales of mood changes: apathy and depression with Hamilton Depression Rating Scale [HDR-S; (53)], disinhibition and hypomania with the Disinhibition Scale and the Mania Scale [DIS-S (54); MAS (55)].

Finally, in accordance with what we did in our previous work (56), “we followed the classification of Starkstein and colleagues (57) who used principal component analysis to subdivide the Awareness of Deficit Questionnaire—Dementia scale [AQD (58)] into four domains taking into consideration the factors loading on each item. One of these factors, identified in terms of impaired awareness in instrumental activities of daily living (AQD_iADL), was designated as factor 1 by the authors. Factor 1 embraces 12 items: ‘recalling the date, orienting to new places, recalling telephone calls, remembering the location of objects at home, understanding conversation and the plot of a movie, keeping belongings in order, handling money, doing mental calculations, remembering shopping lists and appointments, performing clerical work.’ Thus it accounted for most of the variance and also rated as the earliest functional deficit in patients with cognitive impairment” [(56), p. 65]. Since disabilities in instrumental activities of daily living were one of the descriptors of frailty used in the Canadian Study on Health and Ageing (59), we decided to take it into consideration in our analyses.

### Procedures

We implemented a cross-sectional study to explore data collected from our clinical population. Patients were evaluated by performing a neuropsychological assessment during a week in hospital. The participants were assessed in three experimental sessions held 1 day apart and each lasting 1 h, with a view to preventing fatigue and lack of adherence to the tasks.

### Statistical Analysis

We performed statistical analyses using SAS/STAT^®^ 9.3 (60, 61). The *post hoc* power was determined using the formula for sample size with continuous measures through G*Power 3, which is a stand-alone power analysis tool for statistical tests commonly used in social and behavioural research (62). The achieved power with a sample size of 60 was estimated to provide a minimum of 70% power at a 5% level of significance (two-sided) to detect a medium effect (*r*^2^ = 0.2).

To study whether the level of the MPI index could be associated with cognitive and behavioural measurements, we conducted three multiple linear regression analyses adjusted for age, gender, and schooling. Normality assumption distribution of linear regression model residuals was evaluated by means of the Kolmogorov–Smirnov test.

Importantly, the selection of the three models was performed in line with the results obtained in the literature on physical frailty. In particular, frailty has been previously associated with general cognitive decline, impaired global cognition, and memory (5, 8, 18). Moreover, executive function and attention domains have been consistently related to frailty (8–10, 12–20). Finally, the association between physical frailty and the performance at the SART test (8) seems to suggest that pre-frail and frail people might be impaired in response monitoring.

The final selected models considered the MPI as the dependent variable and the following as independent variables:

- Model (1) to address the role of global cognitive functioning and specific cognitive variables (selective attention, episodic memory, language comprehension, and reasoning in the visual modality): ACE-R, MA, BABCOCK, TT and CPM-36;
- Model (2) to study the role of metacognitive executive functions with m-WCST: FCI, GM, MR, and MG;
- Model (3) to investigate the relationship with mood changes, quality of life and awareness of autonomy in instrumental activities of daily living: HDR-S, MAS, DIS-S, QOL-AD, and AQD_iADL.

## Results

Over a 24-month period, 72 patients—complaining of different cognitive deficits and presenting for the first time at the out-dep of our clinics—were evaluated with the overall neuropsychological assessment. After a month, based on CSF analysis results, only subjects with an AD-compatible liquor were included in the experimental sample. As a result, 12 patients were excluded, while 60 subjects were enrolled. Based on the exclusion criterion, 12 patients were excluded, while 60 subjects (M/F = 22/38; mean age ± SD = 69.6 ± 6.8 years) were enrolled (see Table 1 for demographic and clinical data). In particular, 24 MCI due to AD patients according to the CSF analysis, were included in the study. For those patients with major neurocognitive disorders, the CSF diagnosis provided *in vivo* evidence of Alzheimer’s pathology for 36 patients. The neuropsychological assessment reflected the diagnoses made by the CSF (see Table 2 and Table S1 in Supplementary Material), biomarkers and neurological exams. Fifty-six of the 60 patients obtained a CDR score of between 0 and 1 attesting a low level of cognitive impairment.

**Table 1:** Demographic characteristics and frailty evaluation by a comprehensive geriatric assessment.

	Maximum scores	Mean ± SD	Cutoff
**Demographic characteristics**			
Gender (male/female)		22/38	
Age (years)		66.62 ± 6.80	
Schooling (years)		9.28 ± 3.86	
Early cognitive symptoms complaints (months)		31.15 ± 27.82	
Clinical dementia rating scale		0.78 ± 0.44	
**Multidimentional prognostic index**			
Activity of daily living scale	8	5.70 ± 0.53	≥4
Instrumental activity of daily living scale	6	6.63 ± 1.55	≥6
Short portable mental state questionnaire	10	2.46 ± 1.63	≤2
Cumulative illness rating scale—comorbidity index	13	1.43 ± 1.42	
Mini nutritional assessment	30	21.32 ± 3.62	≥24
Exton Smith Scale	20	18.80 ± 1.47	≥15
Polypharmacy		4.12 ± 2.46	
Social condition		Household	
Multidimensional prognostic index	1	0.19 ± 0.12	
**SVAFRA^a^**			
F1		% = 97	
F2		% = 3	

*Wherever there is a normative value, the cutoff scores are given in the statistical normal direction. Cells in grey indicate the absence of a normative cutoff*.

*^a^Scheda di Valutazione dell’Anziano Fragile*.

**Table 2:** Neuropsychological and neuropsychiatric assessment synopsis.

	Maximum scores	Mean ± SD	Cutoff
**Neuropsychological assessment**			
Mini-mental state examination	30	25.02 ± 2.85	≥24
Addenbrooke’s cognitive examination—revised version	100	69.57 ± 12.60	≥82
Attentional matrices	60	30.52 ± 9.91	≥31
Trial making test A	500	110.73 ± 82.92	≤94
Trial making test B	500	297.18 ± 158.12	≤283
Trial making test B-A		194.92 ± 127.11	≤187
Babcock	16	6.22 ± 3.72	≥4.75
Coloured progressive matrices-36	36	22.17 ± 7.26	≥18.96
Token test	36	30.21 ± 3.61	≥32.69
Wisconsin card sorting test % correct answers		52.59 ± 15.44	≥37.1
Wisconsin card sorting test % perseverative errors		34.34 ± 14.95	≤42.7
**Wisconsin card sorting test—metacognitive version**			
Accuracy score		0.03 ± 0.11	
Free choice improvement		−1.17 ± 4.98	
Global monitoring		−20.13 ± 14.29	
Monitoring resolution		0.19 ± 0.24	
Control sensitivity		−0.04 ± 0.58	
Monetary gains		2.37 ± 1.93	
**Neuropsychiatric assessment**			
Quality of life scale in Alzheimer’s disease—patient module	39	17.90 ± 8.01	
Hamilton depression rating scale	67	10.95 ± 7.47	≤7
Disinhibition scale	96	9.40 ± 6.95	≤16.9
Mania scale	44	2.62 ± 3.57	≤15
Awareness of deficit questionnaire—dementia scale for instrumental activity domain	16	3.20 ± 8.22	≤4

*Wherever there is a normative value, the cutoff scores are given in the statistical normal direction. Cells in grey indicate the absence of a normative cutoff*.

### Prevalence of Frailty Status

As regard the CGA evaluation, the patients fell within the lowest MPI range, attesting a low risk of severe prognosis. Considering the SVAFRA index, 97% of all patients were classified as pre-frail, and 3% with medium frailty.

### Association between Frailty and Neuropsychological Variables

After adjusting the analysis for age, gender, and schooling, the MPI index scores were influenced by MR and MONEY in model 2, and by HDR-S, DIS-S, and AQD_iADL in model 3. On the contrary, the level of pre-frailty measured through the MPI index was not influenced by global cognition, memory, language comprehension, non-verbal reasoning (in model 1), or by QOL-AD (in model 3, see Table 3).

**Table 3:** Effect of the independent variables on the multidimensional prognostic index estimated by the univariate and multiple linear regression analyses.

		Crude estimates			Adjusted estimates		
Model	Independent variables	β	*p*	SE	β	*p*	SE
1	Addenbrooke’s cognitive examination—revised version	−0.20	0.18	0.001	−0.03	0.87	0.001
	Attentional matrices	−0.36	0.02	0.002	−0.29	0.11	0.002
	Babcock	−0.05	0.75	0.005	0.09	0.60	0.006
	Token test	−0.12	0.43	0.005	−0.01	0.95	0.005
	Coloured progressive matrices-36	−0.27	0.06	0.002	−0.14	0.44	0.003

	Model significance	*R*^2^ = 0.2056, *F* = 1.42 *p* = 0.2137					

2	Free choice improvement	0.07	0.60	0.003	0.03	0.84	0.003
	Global monitoring	−0.07	0.60	0.001	0.11	0.42	0.001
	Monitoring resolution	0.33	0.02	0.075	***0.37***	***0.01***	0.073
	Monetary gains	−0.21	0.14	0.009	−***0.30***	***0.04***	0.009

	Model significance	*R*^2^ = 0.2863, *F* = 2.46 *p* = 0.0322					

3	Hamilton depression rating scale	0.49	<0.00	0.002	***0.41***	**<*0.00***	0.002
	Mania scale	0.09	0.52	0.005	−0.25	0.07	0.005
	Disinhibition scale	0.50	<0.00	0.002	***0.52***	**<*0.00***	0.003
	Quality of life scale in Alzheimer’s disease	−0.21	0.17	0.002	−0.01	0.91	0.002
	AQ-D scale for instrumental activity domain	−0.14	0.35	0.002	−***0.30***	***0.02***	0.002

	Model significance	*R*^2^ = 0.4980, *F* = 5.46 *p* < 0.0001					

*In multivariable analyses standardised coefficients are adjusted for age, gender, and schooling. Values in bold italics showed a statistically significant association (*p* < 0.05)*.

*AQ-D, Awareness Questionnaire in Dementia*.

The normality distribution assumption of residuals in each model is fulfilled (Kolmogorov–Smirnov test > 0.05).

## Discussion

The aim of this study was to analyse the association among a multidimensional assessment of frailty, executive dysfunction and specific cognitive and behavioural variables using an overall neuropsychological battery. Our findings suggested that pre-frailty was associated with metacognitive executive dysfunction, in terms of action monitoring in MCI-likely due to AD and AD patients. Specifically, we observed a significant association between the MPI index and MR underlying the role of MR, where patients fail to distinguish between correct and incorrect sorts. The results obtained were specific and not influenced by other cognitive functions such as global cognition, memory, language comprehension, and non-verbal reasoning, with the exception of the selective attention task that reached a near significance level. Moreover, taking the MPI scores into account, we observed an involvement of mood changes in terms of depression, apathy, and disinhibition and a reduced awareness of iADL, while the relationship with hypomania was near to the significance value.

Although we considered patients with different degrees of cognitive impairment, our sample was homogeneous in terms of the etiopathogenesis, MPI level of risk, comorbidity with physical illness, somatic complaints, laboratory tests, and the level of mood changes. Moreover, we excluded all patients with neurological diseases other than AD. Most importantly, our attempt to consider these kinds of patients in the same sample was justified by the regression analysis approach we used and by the international guidelines on ageing that consider patients with cognitive impairment to lie on a continuum between MCI and mild AD (28, 63, 64).

Based on the results we obtained, there appear to be no straightforward associations between pre-frailty and specific aspects of neuropsychological functioning such as global cognition, long-term verbal memory, language comprehension, and non-verbal reasoning. As underlined by a review on cognitive impairment and frailty, not all cognitive domains may become impaired simultaneously (7). Although Canevelli et al. (5) have described a possible association between memory and physical frailty, other studies have found that memory does not seem to be clearly associated with frailty [i.e., Ref. (17, 65)]. As far as we are aware, no previous studies have addressed the association between frailty and language comprehension on the one hand, and frailty and non-verbal reasoning on the other (18). Moreover, we found no generalised cognitive impairment as a prerequisite of pre-frailty in our patients. Nevertheless, previously researches have reported a substantial association between frailty and cognitive deterioration (5, 7, 18, 66), although the phenotype model often used in these does not include cognitive function in its definition (1). Despite these evidences—as previously suggested by Robertson and colleagues (7)—statistical analyses of the proposed components of frailty suggest that, while energy, mobility, mood, physical activity, and strength aggregate as one concept, cognitive function does not clearly correlate with this and therefore may not be part of the frailty syndrome (67, 68). Furthermore, a study of AD patients found that 22% had no indications of frailty (69, 70). Indeed, Fougère and colleagues in their review concluded that “it seems most useful, therefore, to treat frailty and cognitive impairment as related but distinct concepts that frequently co-occur” [(71), p. 3].

Notably, the assessment of executive dysfunction should be seen as an important early predictor of cognitive frailty (9), since these problems may appear as the first cognitive changes in MCI patients (72), with concomitant iADL difficulties (73). Indeed, the early identification of executive impairments may be helpful for predicting the long-term functional outcome (74). Interestingly, O’Halloran and colleagues (8) recently stated that pre-frail and frail individuals seem to make more commission errors and omissions than robust subjects on the SART that evaluates the ability to sustain attention for a long time with a response–suppression element (75, 76). This fact suggests that they may be impaired in response monitoring, as we have demonstrated through the association between pre-frailty and action monitoring using the m-WCST. Indeed, the main predictor at this level has been MR, expressed by Koren and colleagues as the correlation between the level of confidence expected from the response and correctness of the sorts in the entire WCST (46–48). In particular, our patients have shown failure to monitor their own performance considering the errors made during task execution, in terms of low metacognitive self-awareness. Moreover, we have found an association between the MPI and monetary gain, which can be interpreted as subjects’ ability to benefit from environmental feedback, anticipate the consequences of their future actions and make decisions accordingly. In addition, we have also found pre-frailty to be associated with an inability to report unsuccessful experiences in iADL through the Awareness Questionnaire in Dementia (AQ-D) (56). In our previous article concerning impaired awareness of deficits in AD and the role of everyday executive dysfunction (56), mood orientation changes, inhibition, self-monitoring, and set shifting appeared to be important skills for awareness of iADL (AQD_iADL) (56). According to this and considering the neural substrate, AD patients with impaired awareness showed reduced activation in the Mid Cingulate Cortex during an event-related fMRI response-inhibition paradigm, compared to subjects aware of their deficits (77).

Through our current results we hypothesise that pre-frailty may arise by a disruption of the comparator mechanisms responsible for monitoring behavioural mood changes and cognitive disturbances (56). If the executive system does not function properly, as frequently described in MCI-likely due to AD and AD patients (78), the comparator mechanism does not detect incongruities between the previous and the actual state of behaviour/performance. This phenomenon has been observed as an inability: (1) to monitor one’s own performance with reference to the impact of errors made during task execution, in terms of a reduction in metacognitive self-awareness; and (2) to relate fruitless experiences in one’s everyday living through the AQ-D.

The few studies investigating the neuropsychiatric factors (79–81) have suggested that “one mechanism underlying the link between frailty and cognition may be due to psychological factors such as mood. Indeed, mood disorders such as depression have been found to be both a risk factor for and a consequence of frailty” [(7), p. 847]. In line with this, we observed a role of HDR-S scores in pre-frailty condition. The HDR-S actually measures mood changes, in terms of apathetic behaviour and depressive mood possibly related to prefrontal dysfunctions (82). This evidence also suggests a possible role of apathy in pre-frailty conditions (83). Interestingly, apathy, disinhibition, and metacognitive executive dysfunction appear to be neurally inscribed in the same network (84–87). The results we obtained proposed the suggestive hypothesis that pre-frailty may be due to a possible dysfunction of the medial prefrontal–ventral striatal network observed throughout action-monitoring disability, mood changes, and reduced awareness of iADL. These findings ask for new neuroimaging investigations and replication in a larger group of patients.

### Limitations Section

The study here presented has been carefully designed and reached its aims; however, some critical aspects have to be outlined. The first aspect regards the tools used to assess frailty, which could represent a possible confounding factor. In particular, pre-frailty is defined by adopting the SVAFRA that may not be considered appropriate for stratifying the frailty status of the older person and it has been rarely adopted for these purposes. Moreover, the MPI was originally conceived as a prognostic index of mortality in the short- and long-term period (37) and a recent international survey among clinical practitioners on the methods to assess frailty in their daily practice (29) did not include MPI as a tool to assess frailty. However, as previously expressed by Bruyére and colleagues (29), frailty is still detected using different instruments and none of them has been able to prevail as superior to the others (29). Second, the results we obtained are not generalisable for patients with different etiopathogenesis other than AD. However, our study is a first attempt to investigate possible association between pre-frailty and neuropsychological variables in a selected patient population, on the basis of liquor examination and may be limited to identify pathogenetic mechanisms of frailty. This is a cross-sectional study aimed to evaluate a possible association between frailty and neuropsychological variables in MCI-likely due to AD and AD patients. A longitudinal study may be the most correct approach to assess for the presence of cognitive disorders many years before the development of frailty itself. Further studies will be important to better characterised this association over time and replicate these findings in a larger group of patients.

## Conclusion

This study represents a first important attempt to extend the frailty issue to neuropsychological correlates taking in consideration a multidimensional approach These findings suggest that the assessment of pre-frailty conditions must be achieved quantitatively by means of a multidimensional approach to clarify the nature and correlates of this multifaceted phenomenon. In particular, the impact of everyday executive dysfunction, early changes in mood and the awareness of iADL disabilities must be considered. Our results benefit from the homogeneity of the experimental sample in terms of etiopathogenetic mechanisms, disease severity (CDR) and functionality in daily life (ADL/iADL). The evaluation of pre-frailty conditions and their neuropsychological correlates is clinically relevant. Indeed, this multifaceted phenomenon has diagnostic, nosological, and prognostic implications that may affect patients’ wellbeing in the continuum from MCI to AD.

## Author Contributions

The study is based on a concept developed by MA who wrote the paper and took part in the review and critique processes as PI. She also organised the study and participated in the statistical analyses (execution and organisation, review, and critique). SP performed the neuropsychological assessment (execution and organisation), participated in the statistical analyses, participated in writing the paper and created the infographics. RR conducted the statistical analyses (execution), participated in interpretation of results and in writing the paper. ER performed the neurological assessment (execution) and took part in the organisation of the study and in the diagnostic phase (organisation and diagnosis). MZ and MB performed the neuropsychological assessment (execution), participated in writing the paper and in drafting the work. DL and IR supervised the neurological assessment, took part in the organisation of the study and participated in writing the paper (organisation, review, and critique). All the contributors gave their approval of this version of the manuscript to be submitted.

## Conflict of Interest Statement

The authors declare that the research was conducted in the absence of any commercial or financial relationships that could be construed as a potential conflict of interest.

## Footnotes

^1^Frailty in the elderly evaluation sheet.

## Supplementary Material

Table S1 CSF assessment synopsis. The results of all subjects are reported.
