# Supplementary material for: Neuropsychological Correlates of Pre-Frailty in Neurocognitive Disorders: A Possible Role for Metacognitive Dysfunction and Mood Changes
Source: Front Med (Lausanne). 2017 Nov 15;4:199. doi: 10.3389/fmed.2017.00199 (PMC5694746; doi:10.3389/fmed.2017.00199)
Supplement: Table S1 — CSF assessment synopsis. The results of all subjects are reported. [file data_sheet_1.doc]

| **Patient (ID)** | **t-tau** | **p-tau** | **AB-42** |
| --- | --- | --- | --- |
| 01 | 140 | 80,2 | 1409,3 |
| 02 | 93,2 | 37,9 | 513,8 |
| 03 | 483 | 109,1 | 794,6 |
| 04 | 99 | 18 | 774 |
| 05 | 101,8 | 46,1 | 1238,4 |
| 06 | 75 | 20,7 | 925,8 |
| 07 | 78,5 | 33,9 | 1097,2 |
| 08 * | - | - | - |
| 09 | 75 | 28 | 431 |
| 10 | 201 | 95 | 1730 |
| 11 | 113 | 38 | 450 |
| 12 | 75 | 28 | 1126 |
| 13 | 75 | 26 | 582 |
| 14 * | - | - | - |
| 15 | 75 | 19 | 987 |
| 16 | 75 | 26 | 494 |
| 17 | - | - | - |
| 18 | 75 | 43 | 1210 |
| 19 | 75 | 29 | 1215 |
| 20 | 121 | 23 | 840 |
| 21 | 83 | 44 | 679 |
| 22 | 109 | 35 | 1183 |
| 23 | 111 | 56 | 258 |
| 24 | 109 | 60 | 357 |
| 25 | 123 | 51 | 553 |
| 26 | 75 | 33 | 1398 |
| 27 | 100 | 54 | 613 |
| 28 * | - | - | - |
| 29 | 111 | 42 | 1132 |
| 30 |  |  |  |
| 31 | 357 | 133 | 627 |
| 32 | 366 | 103 | 405 |
| 33 | 200 | 53 | 511 |
| 34 | 130 | 37 | 609 |
| 35 | 153 | 37 | 715 |
| 36 | 253 | 50 | 710 |
| 37 | 264 | 93 | 716 |
| 38 * | - | - | - |
| 39 | 162 | 56 | 625 |
| 40 | 75 | 23 | 1039 |
| 41 | 75 | 26 | 1222 |
| 42 | 75 | 27 | 978 |
| 43 | 96 | 54 | 2090 |
| 44 | 139 | 73 | 797 |
| 45 | 133 | 42 | 1266 |
| 46 | 143 | 42 | 634 |
| 47 | 110 | 49 | 1145 |
| 48 * | - | - | - |
| 49 | 100 | 32 | 813 |
| 50 | 107 | 53 | 1007 |
| 51 | 75 | 41 | 1101 |
| 52 * | - | - | - |
| 53 | 880 | 133 | 679 |
| 54 | 110 | 50 | 631 |
| 55 * | - | - | - |
| 56 | 75 | 12 | 168 |
| 57 | - | - | - |
| 58 | 75 | 13 | 591 |
| 59 | 181 | 68 | 789 |
| 60 | 75 | 10 | 716 |

* Anatomical contraindications to cerebrospinal fluid tests.
